# Humoral immunity in cancer

**DOI:** 10.1038/s41392-026-02902-2

**Published:** 2026-08-19

**Authors:** Jeanne Leonard, Marie Duhamel, Michel Salzet

**Affiliations:** 1 Univ. Lille, Inserm, CHU Lille, U1192 - Protéomique Réponse Inflammatoire Spectrométrie de Masse - PRISM, Lille, France; 2 Institut Universitaire de France, , ministère de l’Enseignement supérieur, de la Recherche et de l’Innovation, Paris, France; 3 Equipe Labelisée La ligue contre le cancer, Paris, France

**Keywords:** Cancer, Immunology

## Abstract

Humoral immunity driven by B cells, plasma cells, and antibodies has emerged as a major determinant of tumor control, immune escape, and therapeutic response. Across solid tumors, mature tertiary lymphoid structures (TLS) with high endothelial venules, follicular dendritic-cell networks, and T follicular helper cell niches support germinal-center-like reactions, B-cell receptor selection, somatic hypermutation, class-switch recombination, antigen presentation, and local humoral memory. However, B-lineage responses are not uniformly protective. Regulatory B cells, IgA- or IgG4-skewed plasma cells, inhibitory Fc-receptor signaling, chronic immune complexes, and tumor-imposed metabolic stress can suppress cytotoxic immunity and reprogram myeloid compartments. This review synthesizes the mechanisms that determine whether humoral immunity is antitumor or protumor, including TLS maturity, B-cell and plasma-cell states, antibody subclass and tissue geography, Fc glycosylation, Fc-receptor balance, complement context, immunometabolic adaptation, and spatial organization. We also discuss how single-cell, B-cell receptor repertoire, spatial transcriptomic, and spatial proteomic approaches can resolve antigen-driven B-cell selection and distinguish TLS-organized effector niches from diffuse regulatory infiltrates. Finally, we map current and emerging therapeutic strategies, from approved B-cell and antibody-directed agents in hematologic malignancies to TLS-inducing, Fc-engineering, vaccine, and isotype-modulating approaches in solid tumors, and we propose biomarker-guided guardrails for clinical translation. These guardrails are intended to preserve protective TLS-linked immunity while selectively targeting suppressive humoral programs.

## Introduction

Cancer immunology has historically been framed around cytotoxic T-cell surveillance and the therapeutic release of T-cell checkpoints, but this T-cell-centric view is no longer sufficient to explain the full immune architecture of many tumors. B cells, plasma cells, antibodies, complement, and Fc-receptor-bearing effector cells form a second, highly adaptable arm of antitumor immunity. In several solid tumors, tumor-infiltrating B cells are not passive bystanders: they can present antigen, provide costimulatory signals, shape CD4+ and CD8 + T-cell responses, and differentiate into antibody-secreting plasma cells. Clinical and experimental studies now show that the density, differentiation state, and spatial organization of B-lineage cells can influence prognosis and response to immune checkpoint inhibitors, particularly when B cells are embedded in organized tertiary lymphoid structures (TLS). These observations have shifted the field from asking whether B cells are present in tumors to asking which B-cell states are present, where they are located, which antigens they recognize, and how their antibodies engage effector or suppressive circuits in the tumor microenvironment (TME). They also force a distinction between beneficial humoral immunity generated locally within structured immune niches and nonproductive antibody deposition or B-cell infiltration that simply accompanies inflammation.

TLS are ectopic lymphoid aggregates that arise in chronically inflamed tissues and can reproduce key features of secondary lymphoid organs, including high endothelial venules, follicular dendritic-cell networks, T follicular helper (Tfh) cells, germinal-center-like zones, and local plasma-cell differentiation. In tumors, mature TLS can support antigen-driven B-cell receptor diversification, somatic hypermutation, class-switch recombination, and affinity maturation, thereby generating local IgG- and context-dependent IgA-producing plasma cells. These TLS-associated reactions can coordinate with T-cell immunity by sustaining antigen presentation and by recruiting Fc-mediated effector mechanisms such as antibody-dependent cellular cytotoxicity, antibody-dependent cellular phagocytosis, and complement activation. Mature TLS also provide a spatial scaffold in which CXCL13, CCL19/CCL21, IL-21, CD40/CD40L, and lymphotoxin signaling can reinforce Tfh-B-cell crosstalk and maintain humoral memory. The degree of TLS maturation is therefore a functional variable, not only an anatomical descriptor: immature aggregates may recruit lymphocytes without supporting durable germinal-center-like selection, whereas mature TLS can sustain repeated rounds of selection and memory formation. Thus, humoral immunity can amplify a productive tumor-immune cycle rather than merely reflecting the presence of inflammatory infiltrates.

The same pathways can also be co-opted by tumors. Regulatory B cells that secrete IL-10, TGF-beta, or IL-35; IgA+ plasma-cell programs associated with PD-L1 and IL-10; IgG4-skewed responses under chronic antigen exposure; inhibitory Fc-gamma receptor signaling; and soluble or chronic immune complexes can dampen cytotoxic immunity and remodel myeloid cells toward tumor-supportive states. Antibody function therefore depends not only on antigen specificity but also on isotype and subclass, Fc glycosylation, immune-complex geometry, Fc-receptor balance, cytokine milieu, metabolic constraints, and tissue context. A mucosal tumor enriched in IgA-producing cells may require a different interpretation from a non-mucosal tumor dominated by class-switched IgG1/IgG3 plasma cells within mature TLS. Likewise, BCR clonal expansion is informative only when interpreted with somatic hypermutation, class-switching status, phenotype, and spatial localization. This context dependence explains why indiscriminate B-cell depletion may be harmful in some tumors yet rational in others dominated by diffuse regulatory B-cell infiltrates. It also explains why the same therapeutic class, such as checkpoint blockade or Fc-engineered antibodies, can either amplify protective humoral responses or expose patients to autoimmune toxicity if autoreactive B-cell clones are released from tolerance.

Technological advances now make this contextual interpretation possible and define the focus of this review. Bulk B-cell receptor repertoire sequencing can reveal clonal expansion and class switching, but paired single-cell RNA-seq and scBCR-seq connect each clone to a defined transcriptional state, allowing investigators to distinguish germinal-center-like B cells, memory B cells, plasmablasts, plasma cells, and regulatory populations in the same lesion. Spatial transcriptomic and spatial proteomic platforms add the missing anatomical layer by testing whether expanded clones reside inside TLS, at invasive margins, around vessels, or as diffuse infiltrates in immunosuppressive stroma. Together, these tools have enabled TLS maturity metrics and state-resolved B-cell atlases that are beginning to inform patient stratification. We synthesize the determinants that switch humoral immunity from antitumor to protumor, including TLS maturation, B-cell and plasma-cell heterogeneity, BCR clonal expansion, antibody subclass geography, Fc-receptor and complement signaling, immunometabolism, and tumor-specific examples in breast, lung, and brain cancers. We also discuss therapeutic opportunities and safety guardrails, including the timing of B-cell-modulating strategies, the double-edged effects of immune checkpoint blockade on autoreactive B-cell clones, and the need for pretreatment diagnostic panels that distinguish TLS-mature tumors from those in which suppressive humoral programs predominate. By integrating mechanism, spatial context, and clinical translation, the review aims to provide a practical framework for using B-lineage biology as a biomarker and therapeutic axis rather than a single prognostic label.

## Historical perspective on humoral immunity in cancer

**Fig. 1:**
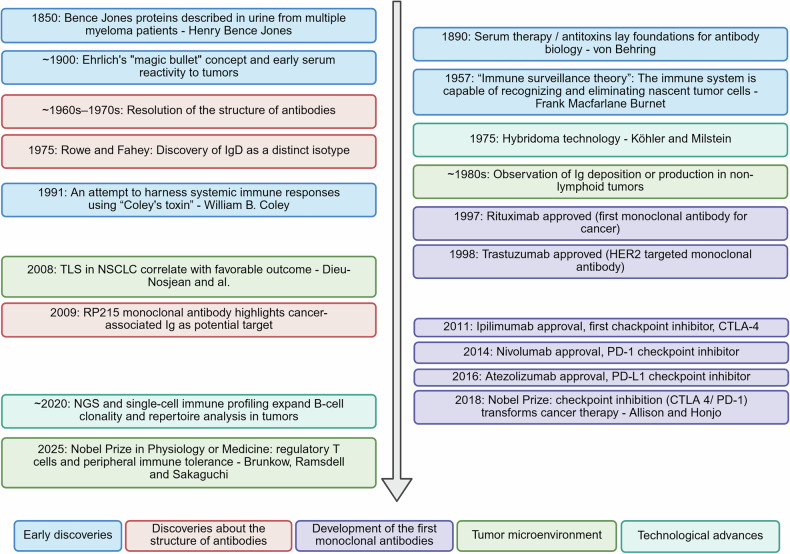
Timeline of selected milestones linking humoral immunity and cancer. The timeline summarizes early observations of immunoglobulin-associated malignancy, the development of serum therapy and infection-based immunotherapy, the conceptual emergence of immune surveillance, the invention of monoclonal antibody technology, the clinical validation of antibody therapeutics, the recognition of TLS and tumor-infiltrating B cells as prognostic and immunotherapy-associated features, and the recent integration of single-cell, BCR repertoire, spatial, and biomarker-guided approaches. The schematic was redrawn manually using non-generative vector graphics and exported as a 300 dpi JPG; no generative-AI image tool was used. Created with BioRender.com

Humoral immunity’s connection to cancer has deep historical roots (Fig. 1). One early link came in 1850, when Henry Bence Jones described abnormal immunoglobulin proteins in the urine of a patient with *mollities ossium* (later recognized as multiple myeloma), a malignancy characterized by monoclonal immunoglobulin production.^1^ Later, in the late 19th century, surgeons like William Coley observed tumor regressions after severe infections. In 1891, Coley began intentionally inducing infections (using “Coley’s toxin”), an early attempt at immunotherapy that leveraged systemic immune responses. However, the specific contribution of antibodies was not fully appreciated until much later.^2,3^ Emil von Behring’s 1890 demonstration that diphtheria can be cured by transferring immune serum marked the birth of passive immunotherapy and laid the foundation for antibody therapeutics.^4,5^ Early 20th-century scientists, including Paul Ehrlich, articulated the “magic bullet” vision that inspired later targeted (including antibody-based) therapeutics.^6^ By the mid-20th century, radiolabeled antibody tracer experiments established that antibodies can be tracked in vivo and paved the way for tumor-targeting strategies, including early demonstrations of tumor-localizing antibodies in cancer models.^7,8^ Naturally occurring ABO isohemagglutinins (anti-A and anti-B) provide a well-characterized example of pre-existing humoral immunity in humans.^9^

In 1957, Frank Burnet proposed the immune surveillance theory, in which the immune system (implicitly including antibodies) can recognize and eliminate nascent tumor cells, a concept pivotal to cancer immunology’s development.^10^ By the 1960s–1970s, key discoveries in immunology laid groundwork: the structure of antibodies was solved, and monoclonal antibody technology was developed in 1975 by Köhler and Milstein. The invention of hybridoma technology revolutionized our ability to produce monoclonal antibodies,^11,12^ directly enabling the development decades later of therapeutic antibodies like rituximab. In 1975, another landmark was the discovery of IgD as a distinct isotype by Rowe and Fahey. By the 1980s, with improved serological and imaging techniques, researchers noted immunoglobulin deposition or production in non-lymphoid tumors, although it remained controversial. The late 20th century saw therapeutic dividends of humoral immunity: monoclonal antibodies such as trastuzumab (anti-HER2) and rituximab (anti-CD20) entered clinical practice, validating Ehrlich’s idea of “magic bullet” antibodies against cancer. These successes reinvigorated interest in the roles of B cells and antibodies within tumors themselves.

## Components and mechanisms of humoral immunity in the tumor microenvironment

Humoral immunity in cancer involves a cast of cells, molecules, and interactions that together influence tumor fate. Key components include B lymphocytes (with various differentiation states), the antibodies (immunoglobulins) they produce, and complementary elements such as the complement system and Fc receptors on effector cells.

### B cells and plasma cells in tumors

B cells are the lymphocytes responsible for antibody production. They originate from bone marrow progenitors, undergo gene rearrangement to create a unique B-cell receptor (surface Ig), and then populate secondary lymphoid organs. In cancer, different B-cell subsets can be found in or around tumors, each with distinct roles.^13^

#### Naïve B cells

These are mature B cells that have not yet encountered their specific antigen. They circulate through lymph nodes and can be recruited to tumors by chemokine gradients and stromal interactions.^14^ Naïve B cells can be activated upon recognizing tumor antigens (directly or presented by dendritic cells) and receiving T-cell help, initiating anti-tumor antibody responses.^15–17^

#### Germinal center (GC) B cells

Upon antigen encounter in the presence of T follicular helper cells, B cells can form germinal centers (in lymph nodes or tumor-associated TLS) where they proliferate and undergo somatic hypermutation and class-switch recombination.^15–18^ GC B cells in tumors indicate an ongoing local antibody refinement process; their presence often correlates with high-affinity anti-tumor antibodies and improved antigen presentation to T cells.^16^ Markers like BCL6 and AID (activation-induced deaminase) denote GC activity.^19^

#### Memory B cells

These are long-lived B cells that have been previously activated and can rapidly respond upon antigen re-exposure.^20^ In cancers, memory B cells (typically CD27⁺ in humans) can reside in TLS or bone marrow.^21^ They can reflect prior exposure to tumor antigens (e.g., from an earlier stage lesion or cross-reactive pathogen). Memory B cells can quickly differentiate into plasma cells upon sensing tumor antigen again, thus contributing to an accelerated humoral response.^22^ Tumors with a high density of memory B cells in TLS often show enhanced response to checkpoint blockade therapy (because these B cells present tumor antigen to T cells and produce immunostimulatory cytokines).^23^

#### Plasma cells (antibody-secreting cells)

These terminally differentiated B cells are devoted to antibody production.^24^ Plasma cells can be found at the tumor invasive margin or within TLS, identified by markers like CD138 (syndecan-1) and IRF4/PRDM1 (Blimp-1). They secrete large quantities of immunoglobulins, which can target tumor antigens (if the plasma cell was tumor-specific) or irrelevant antigens (e.g., if polyclonal activation occurred). High infiltration of plasma cells in some cancers (e.g., melanoma, colorectal) has been associated with better patient survival and immunotherapy response, due to the presence of tumor-specific antibodies and sustained local immune activation.^25–27^ However, plasma cells can also secrete immunosuppressive isotypes (like IgA or IgG4) that modulate myeloid cells.^28–30^ In glioblastoma, a recent study found plasma cells are aberrantly enriched and support tumor stem cells via secreted IgG,^31^ a striking example we discuss later.

#### Regulatory B cells (Bregs)

This subset, often defined functionally rather than by a single marker, produces anti-inflammatory cytokines (IL-10, IL-35, TGF-β) and can dampen anti-tumor immunity. Bregs can express markers like CD5 or CD24⁺CD38⁺ (in humans) and typically exhibit an IgM⁺ memory phenotype.^32^ They inhibit effector T cells and NK cells and promote T regulatory cell (Treg) development.^33^ Tumor-educated Bregs have been identified in several cancers (breast, ovarian) where they facilitate metastasis through IL-10 and TGF-β release.^34,35^ Elevated IL-10-producing B cells in patients often correlate with poor immune responses. Notably, some Bregs also secrete IgG4 antibodies that switch macrophages toward an M2 immunosuppressive phenotype.^36,37^

#### B1 cells and natural antibodies

B1 cells are an innate-like B cell subset (mainly in pleural/peritoneal cavities) that produce natural IgM antibodies without obvious antigen stimulation.^38^ They can migrate to tumors or draining lymph nodes and provide low-affinity IgM that can initiate complement activation on tumor cells.^39^ Some natural IgM have tumor-binding reactivity (for example, recognizing altered self-lipids or sugars on tumor cells) and can induce complement-mediated cytotoxicity40. However, natural antibodies can also form immune complexes that paradoxically dampen immune responses via Fc receptors on macrophages.^40^ B1 cells are less studied in human tumors but are believed to contribute to the antibody pool within some TLS.^41^

B lymphocytes are frequently found infiltrating solid tumors, where they can organize into structures resembling lymphoid follicles. When such aggregates are well-developed with germinal centers and high endothelial venules, they are termed TLS. TLS indicate an ongoing local immune response with B cell activation and antibody production. Many tumors such as melanoma, lung cancer, colorectal carcinoma, and breast cancer exhibit TLS at their periphery or stroma, and their presence is generally correlated with improved patient survival and better response to immunotherapies.^42–45^ Within TLS, B cells interact with T follicular helper cells, dendritic cells, and other immune cells to undergo affinity maturation and class switching, akin to lymph nodes. The result is local generation of plasma cells that secrete high-affinity antibodies against tumor antigens. Indeed, multiple studies have confirmed the presence of isotype-switched IgG and IgA antibodies at tumor sites, often produced by TLS-resident B cells.^46^ High densities of TLS with germinal center B cells and plasma cells often correlate with improved prognosis (as in melanoma, non-small cell lung cancer, and sarcomas).^26,47–49^ Conversely, diffuse infiltration by IL-10⁺ B cells or enrichment of IgA⁺ plasma cells (which can drive immune suppression via FcαRI on myeloid cells) is linked to poor outcomes in some settings.^28,50^ Thus, the net effect of B cells in each tumor depends on the balance of effector B cells vs. regulatory B cells and the spatial organization of these cells (e.g., contained in TLS vs. dispersed). For example, in breast cancer, intratumoral IgG and IgA levels are high and associated with the presence of TLS and antigen-driven B cell responses. In melanoma, antibodies specific to melanoma antigens have been isolated from tumor sites, demonstrating that B cells recognize tumor antigens and mount responses in situ.

By serving as antigen-presenting cells (APCs), B cells can also boost anti-tumor T cell responses. Activated B cells (or plasma cells) in tumors can present tumor-derived peptides on MHC II molecules to CD4⁺ T cells, providing critical co-stimulation that sustains cytotoxic T lymphocyte activity.^51^ This B-T cell cooperation in TLS has been reported to generate a powerful dual immune attack: B cells provide tumor-specific antibodies and concurrently help activate T cells that can directly kill tumor cells. Tumors with robust TLS often display such coordinated B and T cell activity, explaining their better prognosis.^49^ Conversely, an absence of B cells or TLS can denote a more immunologically “cold” tumor, often less responsive to immunotherapy.

### Antibodies

Effector Functions and Subclass Differences: Antibodies produced in the context of cancer can exert several effector functions, including complement activation, opsonisation for phagocytosis, and engagement of Fc receptors on effector cells leading to cytotoxicity. These functions depend on antibody isotype/subclass and Fc glycosylation.^52–54^ IgM is a potent initiator of the classical complement pathway, and IgM bound to tumor antigens can trigger complement-mediated injury through C1q engagement and downstream membrane attack complex formation.^55,56^ IgG is the dominant class-switched isotype in most solid-tumor TLS responses, and IgG1/IgG3 subclasses bind C1q and activate Fcγ receptors (including FcγRIII/CD16 on NK cells and FcγRI/CD64 on macrophages) to promote ADCC/ADCP and complement engagement.^53,54,57–59^ IgG4 is functionally Fc-silent for complement and has weak affinity for activating FcγRs; increased IgG4 has been reported in several tumor settings and can competitively dampen IgG1-mediated effector functions.^37,60–62^ IgA is prominent in mucosal tumor contexts and engages FcαRI/CD89 on neutrophils and monocytes/macrophages; cross-linked IgA immune complexes can induce strong neutrophil-mediated tumor cell killing, whereas monomeric IgA in regulatory cytokine milieus can bias FcαRI toward inhibitory ITAM signaling.^63–66^ Accordingly, antibody function in tumors is shaped by isotype/subclass composition, Fc receptor balance, immune-complex geometry, and the surrounding cytokine milieu.

### Complement

When antibodies (especially IgM or IgG1) bind to antigens on a tumor cell surface, the C1q component of complement can attach to the antibody’s Fc region, initiating the classical complement cascade.^55,56^ This leads to opsonization of tumor cells with C3b (promoting phagocytosis by macrophages) and formation of membrane attack complexes that can directly lyse tumor cells. Complement activation within tumors can have contradictory effects, on one hand contributing to tumor cell killing, but on the other, excessive complement activation can drive chronic inflammation that supports tumor growth.^67^ Indeed, some cancers upregulate complement regulatory proteins (like CD55, CD59) to protect themselves from complement-mediated lysis, highlighting a tug-of-war between humoral effectors and tumor immune evasion strategies.^54^

### Fc receptor-mediated cytotoxicity

The Fc regions of IgG or IgA antibodies bound to tumor cells can be recognized by Fc receptors on innate immune cells.^57,58,68^ NK cells bearing FcγRIII (CD16) can bind IgG1/IgG3-coated targets and release perforin/granzymes to kill the target.^59^ This is the classic mechanism of ADCC exploited by many therapeutic antibodies (e.g., rituximab-coated B cells are lysed by NK cells).^69^ Macrophages and neutrophils express FcγRII and FcγRIII for IgG, and FcαRI for IgA; when they engage antibody-coated tumor cells, they can mediate phagocytosis or ADCC as well.^70,71^ In addition, FcγRI (CD64) on macrophages binds IgG with high affinity and can trigger pro-inflammatory cytokine release and phagocytic activity against opsonized tumor cells.^72^ These interactions illustrate how antibodies serve as a bridge connecting tumor cells to immune effector cells.

### Subclass nuances (IgG4 and IgA)

Tumors often create a cytokine milieu (e.g., IL-4, IL-10) that can skew class switching towards IgG4, especially under chronic antigen exposure. IgG4, while a minor fraction of circulating Ig in healthy individuals, is frequently increased in several cancers (including pancreatic cancer, cholangiocarcinoma, and melanoma) and can co-localize with IL-10–rich regulatory niches.^73,74^ Functionally, IgG4 can compete with cytotoxic IgG subclasses for antigen binding without efficiently recruiting ADCC or complement, and it can undergo Fab-arm exchange, further limiting immune-cell cross-linking.^37,60,61^ Clinically, elevated IgG4 levels have been associated with worse outcomes in some cohorts,^37,62^ consistent with an immune-escape phenotype. IgA in tumors is likewise context dependent. IgA⁺ plasma cells are abundant in subsets of tumors (e.g. lung adenocarcinoma, gastrointestinal cancers and melanoma) and can co-express immunosuppressive markers such as IL-10 and PD-L1.^16,29,30,75,76^ In several mouse models, IgA⁺ cells suppress anti-tumor CD8⁺ T cells; for example, Shalapour et al. ^77^ showed that IgA⁺ plasma cells in inflammation-driven liver cancer produce IL-10 and PD-L1, blunting local CD8⁺ T cells.^77,78^ Mechanistically, the IgA Fc receptor FcαRI/CD89 can deliver inhibitory ITAM signaling (ITAMi) when engaged by monomeric IgA, but it triggers potent activating effector functions when cross-linked by IgA immune complexes.^64^ Thus, high local concentrations of monomeric IgA in regulatory cytokine environments can favor inhibitory FcαRI signaling and functional “Fc competition” with IgG, whereas antigen-specific IgA immune complexes can recruit neutrophils for cytotoxicity in appropriate contexts.^65,66,79–81^ Accordingly, IgA is not intrinsically immunosuppressive; its net effect depends on immune-complex context, FcαRI engagement geometry, and the surrounding cytokine milieu.

Isotype geography should also be interpreted in light of tissue origin. Mucosal and mucosa-associated tumors, including gastrointestinal cancers and subsets of lung cancer, are more likely to contain IgA-rich plasma-cell programs because local cytokines, microbiota-linked inflammation, and mucosal imprinting favor IgA class switching. In contrast, non-mucosal solid tumors such as melanoma, renal cell carcinoma, sarcoma, and many breast cancers more often show IgG-dominant class-switched TLS responses. This anatomical distinction does not define function by itself: antigen-specific polymeric or immune-complexed IgA can recruit neutrophils and mediate cytotoxicity, whereas monomeric or regulatory IgA in IL-10/TGF-beta-rich niches can suppress effector immunity. The subclass discussion should therefore combine tissue origin, antigen specificity, immune-complex geometry, and Fc-receptor context.^63–66,77,79–81^

Antibody subclass is only one layer of functional tuning. Fc glycosylation is another major determinant of effector activity. For canonical IgG, the conserved Fc N-glycan at Asn297 shapes FcγR binding and downstream ADCC/ADCP, with afucosylated and galactosylated glycoforms generally enhancing activating FcγR engagement, whereas increased sialylation is often linked to anti-inflammatory properties.^82^ Variable-region N-glycans can also occur but are not universal features of tumor immunity. Importantly, O-glycosylation is characteristic of IgA/IgD (and rare in IgG, largely limited to specific hinge contexts such as IgG3), and should not be inferred as a generic hallmark of tumor antibodies without direct glycoproteomic confirmation. In later sections, we therefore separate consensus Fc glycoimmunology from less-validated reports of atypical glycans in tumor-associated Ig species.

**Fig. 2:**
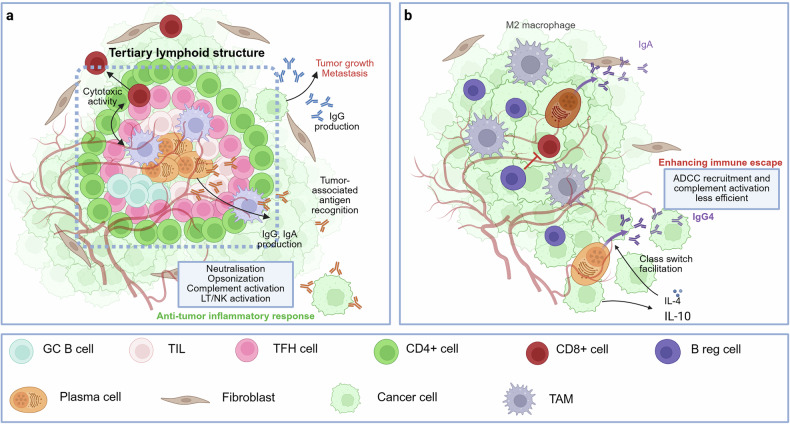
Context-dependent humoral immunity in tumors. **a** TLS-mature antitumor niches contain high endothelial venules, follicular dendritic-cell networks, Tfh cells, germinal-center-like B cells, memory B cells, CD8+ T cells, and plasma cells that support antigen presentation, class switching, antibody production, ADCC, ADCP, and complement. **b** TLS-poor or chronically inflamed regulatory niches can contain Bregs, IgA+ or IgG4+ plasma-cell programs, IL-10/TGF-beta/PD-L1 signaling, chronic immune complexes, Tregs, and M2-like tumor-associated macrophages that suppress CD8+ T cells and remodel myeloid compartments. Contextual switches include TLS maturity, isotype/subclass distribution, Fc-receptor balance, cytokine milieu, antigen persistence, tissue origin, and metabolic stress. The schematic was redrawn manually using non-generative vector graphics and exported as a 300 dpi JPG; no generative-AI image tool was used. Created with BioRender.com

In summary, the humoral components (B cells, plasma cells, antibodies, complement) can be potent mediators of tumor cell killing or facilitators of tumor immune evasion. Their net effect in each cancer is context-dependent. In immune “hot” tumors with TLS, B cells and antibodies often contribute to tumor control, working in concert with T cells. In contrast, in immune “cold” or chronically inflamed tumors, humoral elements can skew toward tumor promotion, exemplified by suppressive IgA plasma cells or blocking IgG4 antibodies. These themes will be elaborated as we examine the determinants that switch humoral immunity from antitumor to protumor (Fig. 2).

## Context-dependent Humoral Immunity in Tumors

### TLS maturation governs the quality of local antibody responses

Mature TLS, characterized by follicular dendritic cell (FDC) networks, high endothelial venules (HEV), and GC-like zones with T follicular helper (Tfh) cells, support robust class-switch recombination and affinity maturation, yielding high-affinity, class-switched IgG (and context-dependent IgA) with antitumor potential.^21,83^ In contrast, immature TLS lacking organized FDC/Tfh niches limit B-cell activation, resulting in restricted somatic hypermutation and narrower Ig repertoires.^48,84^ Multiple studies link mature TLS density to greater BCR diversity and clonal expansions and to improved checkpoint inhibitor responses, whereas diffuse IL-10 + /IgA+ B-cell infiltrates outside TLS correlate with immunosuppression.^27,47,85,86^ TLS ontogeny is steered by CXCL13 and CCL19/CCL21 gradients and LTβR/FDC signaling; experimental TLS induction via these axes enhances local B-cell immunity in models.^87^

Mechanistically, mature TLS are sustained by reciprocal positive-feedback loops rather than by lymphocyte accumulation alone. CXCL13 produced by stromal cells, follicular dendritic cells, Tfh cells, and activated B cells attracts CXCR5 + B cells and Tfh cells into follicular niches, while lymphotoxin-beta receptor signaling stabilizes follicular dendritic-cell networks and high endothelial venules. Within this scaffold, Tfh-derived IL-21 and CD40L promote B-cell proliferation, AID induction, class-switch recombination, somatic hypermutation, and differentiation into plasma and memory B cells. Activated B cells, in turn, maintain Tfh-cell positioning and cytokine production through antigen presentation and costimulatory signaling, closing a CXCL13-IL-21 loop that supports germinal-center-like activity and local humoral memory.^21,27,83,85,88,89^

This functional view explains why TLS maturity is clinically more informative than TLS presence alone. Immature aggregates may recruit lymphocytes but fail to generate sustained clonal selection, whereas mature TLS provide a local site for repeated antigen sampling, affinity maturation, and recall responses. Persistent tumor antigen can therefore maintain a reservoir of memory B cells and long-lived or continuously replenished plasma cells in the TME, supporting durable antibody production and antigen presentation when the tumor is challenged by chemotherapy, vaccination, or immune checkpoint blockade.^21,47,83,85^

### Evidence layers and experimental systems

**Fig. 3:**
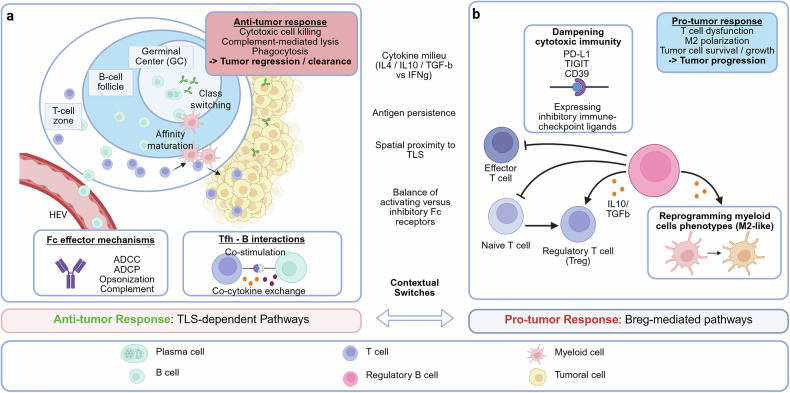
Antitumor and protumor humoral switches in the tumor microenvironment. **a** Antitumor switching is favored by CXCL13/CCL19/CCL21 gradients, HEV formation, TLS maturation, IL-21 and CD40L-driven Tfh-B-cell crosstalk, somatic hypermutation, class-switch recombination, clonal selection, and productive IgG1/IgG3 or cytotoxic IgA immune complexes that drive tumor opsonization, ADCC, ADCP, and complement. **b** Protumor switching is favored by chronic antigen exposure, IL-10/TGF-beta-rich niches, diffuse Bregs, suppressive IgA+ or IgG4+ plasma-cell states, Fc-gamma receptor IIB dominance, Fc-alpha receptor I inhibitory ITAM signaling, T-cell suppression, M2-like myeloid polarization, immune escape, and tumor progression. The distinction between antitumor and protumor pathways was enlarged for legibility in print and digital formats. The schematic was redrawn manually using non-generative vector graphics and exported as a 300 dpi JPG; no generative-AI image tool was used. Created with BioRender.com

Mouse models and perturbation studies are essential to establish causality for specific humoral programs. For example, IgA⁺ regulatory plasma cells induced by chronic inflammation can suppress CD8⁺ T cells via IL-10/PD-L1 and accelerate tumor growth, whereas experimental induction of TLS-like structures can enhance local priming and improve response to immune checkpoint blockade in selected settings. At the same time, species differences in Fc receptor biology, microbiota, and inflammatory context mean that mechanistic findings from mice must be triangulated with human cohort, spatial, and repertoire evidence before being generalized across cancers. (Fig. 3)

## High-throughput analyses of B cells and antibodies in cancer

The complexity of humoral responses in cancer has driven the adoption of high-throughput technologies to profile B cells and antibodies on a broad scale. Modern sequencing and proteomic tools allow researchers to dissect B-cell repertoires, track clonal evolution, and identify antibody specificities within tumors at an unprecedented resolution. Here we highlight key technological advances, particularly single-cell BCR sequencing (scBCR-seq),^90^ repertoire sequencing, and related multi-omics, and discuss how they are advancing our understanding of B cell function in cancer (Table 1).

**Table 1:** Determinants of antitumor versus protumor humoral immunity in cancer (with evidence grading)

Determinant / axis	Antitumor context	Protumor context	Evidence tier
**TLS organization and maturity (HEV+, FDC network, GC-like zones)**	Local affinity maturation, class switching, and coordinated antigen presentation supporting CD8+ T cells and Fc-effector pathways	Absent/immature TLS; diffuse B cells with limited affinity maturation; regulatory niches dominate	A
**Intratumoral B-cell state**	GC-like and memory-like B cells; APC function and cytokines supporting T-cell priming	Regulatory B cells (IL-10/TGF-β/IL-35; PD-L1) and extrafollicular skewing that dampens cytotoxicity	A–B
**Plasma-cell state and isotype**	Class-switched IgG1/IgG3 plasma cells; opsonization and effector recruitment	IgA+ regulatory plasma cells (often IL-10/PD-L1) and/or plasma-cell programs linked to immune suppression	A–B
**Antibody subclass balance**	IgG1/IgG3 dominance, enabling ADCC/ADCP and complement	IgG4 skewing with Fc-silent/competitive binding and Fab-arm exchange, reducing effector function	B
**Fc-receptor landscape**	Activating FcγRs (e.g., FcγRIII/CD16 on NK cells; FcγRI/CD64 on macrophages) and pro-inflammatory signaling	Inhibitory FcγRIIB dominance and/or ITAMi-biased signaling that blunts activation	A–B
**Immune-complex and complement context**	Productive immune complexes promote opsonization, phagocytosis, and complement-mediated injury to tumor cells	Chronic/soluble immune complexes and complement dysregulation that reprogram myeloid cells or promote inflammation without clearance	B
**Fc glycosylation (canonical IgG Asn297)**	Glycoforms that enhance activating FcγR binding (e.g., afucosylated, galactosylated) and effector potency	Anti-inflammatory glycoforms (e.g., increased sialylation) and/or glycan patterns associated with regulatory milieus	B

Evidence tiers: A, replicated across independent cohorts/labs with cell-type resolution in patient tissue; B, supported in patient samples but limited replication and/or mainly preclinical; C, largely cell-line/preclinical or indirect evidence

### B-cell receptor (BCR) repertoire sequencing and clonality analysis

High-throughput sequencing of immunoglobulin heavy (IGH) and light chain genes from tumors or blood can capture the diversity of B-cell clones present. In B-cell malignancies, this approach is used to identify the clonal IGH sequence and monitor minimal residual disease by sequencing the IGH over time.^91^ In solid tumors, repertoire sequencing of tumor-infiltrating B cells or even the bulk tumor can reveal whether there are expanded B-cell clones (which would indicate an antigen-driven response). Several studies in 2018–2021 applied next-generation sequencing to TIL-B cells in cancers like melanoma and lung cancer, finding clonal expansions of B cells in many tumors, often corresponding to those forming TLS. Sequenced BCRs from melanoma TLS and found oligoclonal populations with evidence of somatic hypermutation, implying ongoing antigen selection. Repertoire sequencing can also compare the B-cell populations in the tumor versus peripheral blood of the same patient.^92,93^ This has shown that often the tumor harbors unique clones not abundant in blood, emphasizing the localized nature of the anti-tumor B cell response (driven by tumor antigens not encountered elsewhere). In some cases, identical BCR sequences have been found in separate metastases of the same patient, suggesting a common B cell clone homed to multiple tumor sites (as seen in ovarian cancer by Kroeger et al. 2016).^94^ Such tools also detect class-switching events; for instance, a clone can be present as IgM in blood but as IgG in the tumor, indicating that class-switch recombination occurred in the TLS within the tumor. For clonality assessment in suspected lymphomas, a technique called BIOMED-2 PCR (a standardized multiplex PCR for Ig/TCR genes) was widely used.^95^ Now, high-throughput sequencing provides more depth, detecting even minor B-cell clones.^96^

BCR clonal expansion should therefore be interpreted as a functional metric rather than only as a marker of B-cell presence. Expanded clones with high somatic hypermutation, convergent sequence features, class-switching to IgG1/IgG3 or context-appropriate IgA, and localization within TLS are consistent with antigen-driven selection and engaged effector function. In contrast, unmutated or weakly mutated clones, diffuse localization, IgG4 or regulatory IgA skewing, and association with IL-10/PD-L1 programs may indicate bystander activation or suppressive humoral niches. Pairing clonotype, SHM burden, isotype, phenotype, and spatial localization is therefore essential to distinguish productive effectors from regulatory or irrelevant populations.^90,92–105^

### Single-Cell RNA-seq and BCR-seq (scBCR-seq)

The advent of droplet-based single-cell sequencing (e.g., the 10x Genomics platform) allows simultaneous capture of the transcriptome of individual cells and the paired BCR heavy-light chain sequences of B cells.^99^ scBCR-seq has been revolutionary in dissecting B-cell states in tumors. With scRNA-seq, we can cluster B cells into subsets (naïve-like, germinal center-like, plasma cells, regulatory B cells) based on gene expression, and scBCR-seq links each cell’s antibody sequence to its phenotype. One application in cancer is identifying expanded B cell clones and their isotype within tumors. For instance, Bod et al. performed scRNA/BCR-seq on melanoma tumors and identified checkpoint-regulated B-cell programs relevant to antitumor immunity.^100^ In early-stage lung adenocarcinoma, Hao et al. identified tissue-resident and clonally expanded B-cell states with somatic hypermutation and checkpoint features such as PD-1, consistent with chronic activation and local adaptation.^101^ Crucially, scBCR-seq allows tracking clonal evolution: one can sometimes see a family of B cells all descended from a common ancestor, with some still expressing IgM and others having switched to IgG or IgA, all within the tumor. This mirrors what happens in germinal centers. In cancer, it demonstrates that a robust immune response is ongoing. Additionally, by comparing scBCR from pre- and post-treatment tumor samples, one can see expansion of certain B-cell clones after immunotherapy, indicating those clones responded to treatment (or new clones emerging in relapse). This was shown in a study of melanoma patients on checkpoint inhibitors: responders had an increase in intratumoral B-cell clonality and new Ig class-switched clones after therapy, whereas non-responders did not.^47^ Another high-throughput angle is spatial transcriptomics combined with BCR analysis^102^: for example, researchers have visualized TLS within tumors and confirmed that B cells inside TLS express higher levels of activation-induced genes and have distinct clones compared to dispersed B cells in the tumor parenchyma.^106–108^

### Spatial multi-omics and niche-resolved humoral states

Spatial multi-omics is now essential for resolving whether tumor-infiltrating B cells participate in organized TLS or exist as diffuse regulatory infiltrates. Spatial transcriptomics can map chemokines, Tfh markers, plasma-cell programs, and myeloid niches, while spatial proteomics can quantify Fc receptors, checkpoint ligands, cytokine receptors, and cell-cell proximity at single-cell resolution. When paired with scRNA-seq and scBCR-seq, these platforms can connect each B-cell clone to its isotype, somatic hypermutation burden, transcriptional state, and anatomical niche. This integrated framework refines TLS maturity metrics and avoids overinterpreting bulk B-cell abundance or antibody deposition as evidence of productive humoral immunity.^102–105,109^

Methods such as proteogenomic mapping and mass spectrometry can identify immunoglobulin proteins directly in tumor tissues and patient fluids.

In sum, scBCR-seq and repertoire sequencing provide a comprehensive picture of B cells in cancer, revealing whether B cells are mounting coordinated responses, which classes of antibodies are being produced, and how these clones can adapt or be shaped by the tumor microenvironment. These approaches strongly support the existence of active humoral immune microenvironments in many solid tumors. They also aid in identifying antibody targets, because paired repertoire and transcriptome workflows can prioritize expanded clones for antibody reconstruction and functional testing.^97,98^ For example, if a particular B cell clone is highly expanded, one can infer that its antigen (the target of its antibody) is an important tumor antigen, a candidate for vaccine or antibody therapy development. Indeed, there are efforts to clone antibodies from tumor-infiltrating B cells and test their reactivity; some have been found to bind tumor cell surfaces or tumor antigens like cytokines.^108,110,111^ Consistent with these findings, large-scale single-cell and multi-omics studies have recently mapped intratumoral B cell landscapes across multiple cancers. These pan-cancer analyses reveal an extraordinary heterogeneity of B cells, identifying numerous subsets (e.g., germinal center-like, naïve, memory, plasma, regulatory) within tumors. Notably, B cells following a germinal center-like (follicular) differentiation path (often residing in TLS) are associated with improved immunotherapy responses and survival, whereas those in an extrafollicular trajectory correlate with poorer outcomes. Such insights underscore that the functional balance of humoral immunity (protective vs. suppressive) varies not only between patients and cancer types, but also according to the predominant B cell states within tumors. Taken together, it is becoming clear that B cells are not bystanders but active participants in the tumor-immune dialog, and we finally have the tools to listen in detail.

### Pan-cancer single-cell resources and clinical associations

Recent pan-cancer single-cell resources resolve TIL-B diversity and clinical associations. Fitzsimons and collaborators delineated ~10 recurrent B and plasma cell states across cancers; GC-like B cells and class-switched IgG⁺ plasma cell states co-varied with TLS signatures and favorable outcome in multiple cohorts.^103^ Ma and collaborators combined scRNA-seq with paired BCR-seq to map clonal expansion and somatic hypermutation trajectories within tumors, supporting antigen-driven selection and isotype-specific niches in TLS versus diffuse stroma.^104^ Yang and collaborators integrated data from 649 patients across 19 cancer types to map phenotypically distinct B-cell states with therapy-relevant implications.^105^ These atlas-level observations are consistent with tumor-focused studies linking B cells and TLS to survival or immunotherapy response in specific contexts such as sarcoma and melanoma.^27,47,112,113^ Importantly, most atlas-derived links between B-cell states and outcome/response are associative; causal claims require orthogonal validation (e.g., spatial localization, functional perturbation in model systems).

## Humoral immunity across cancer types: breast, lung, and brain cancers

While general principles apply, the role of humoral immunity can vary notably between cancers such as breast, lung, and brain tumors:

### Breast cancer

Breast tumors, especially the highly immunogenic triple-negative subtype, often display TLS and B-cell infiltrates.^110^ Multiple studies report that a “B-cell gene signature” (indicative of active humoral responses) correlates with better survival and, in some settings, pathological complete response to chemotherapy or checkpoint blockade.^49,114^ Tumor-reactive IgG and IgA antibodies against tumor-associated antigens (e.g., NY-ESO-1, MUC1) have been detected in patients,^110,115^ and IgG⁺ tumor-infiltrating B cells can show features consistent with germinal-center reactions and affinity maturation.

### Lung cancer

Lung cancers (both non-small cell and small cell) can harbor substantial B-cell infiltration and TLS, and the presence of intratumoral B cells/plasma cells, particularly when organized into TLS, often correlates with better survival and improved responses to PD-1 blockade.^116^ In lung squamous cell carcinoma, tumor-infiltrating B cells can class-switch to IgG and IgA, and antibodies have been reported to recognize tumor antigens (e.g., LAGE-1, TP53, NY-ESO-1).^117^ In parallel, immunoregulatory B-cell states (Bregs) have been described,^118^ with IL-10/TGF-β production and checkpoint-ligand expression that can weaken cytotoxic immunity and support pro-tumor myeloid/Treg programs^119^; in mice, Stat3-expressing Bregs were implicated in lung metastasis.^35^ B-cell recruitment can be promoted by CXCL13 in the lung TME,^120^ and single-cell analyses highlight diverse B-cell states spanning antibody-secreting plasmablasts and exhausted, IL-10-producing B cells.^121^ Consistent with context-dependent antibody biology, IgG4 enrichment has been reported in some thoracic tumor settings.^30^

### Brain tumors

The immune environment of the central nervous system (CNS) is shaped by the blood–brain barrier (BBB), microglia, and compartmentalized lymphatic drainage.^122–126^

In glioblastoma (GBM) and many brain metastases, BBB integrity is frequently compromised by neo-angiogenesis and inflammation. As a result, vascular leak can permit serum proteins including immunoglobulins to accumulate within tumor tissue; therefore, the presence of Ig in CNS tumors does not, by itself, imply local antibody production.

Multiple studies document intratumoral B cells and plasma cells (and, in some contexts, TLS-like aggregates), which can represent a substantial local source of antibody in CNS tumors. Functionally, B-cell/plasma-cell programs in GBM have been reported to include immunoregulatory features (e.g., PD-L,^28^ IL-10, TGF-β),^127,128^ and plasma cells have been linked to glioma stemness via antibody-dependent engagement of an FcγRIIA-AKT-mTOR axis.^127^ Together, BBB leak plus infiltrating plasma cells can plausibly explain many antibody-associated phenotypes observed in CNS tumors.

Overall, these examples across cancer types reinforce that humoral immunity’s impact is highly context-dependent. Across multiple solid tumors, the presence of mature TLS is repeatedly associated with GC-like B cells, class-switched plasma cells, and more favorable outcomes and/or improved responses to immune checkpoint blockade (Table S1). Conversely, some tumor milieus skew toward immunoregulatory B-cell/plasma-cell programs (e.g., Bregs, IgA+ regulatory plasma cells, IgG4-biased responses) that can suppress cytotoxic immunity and remodel myeloid compartments. Pan-cancer single-cell atlases provide an important associative map of B-cell states and clonal architectures, but causal language should be avoided unless supported by functional perturbation studies.

## Molecular signal transduction in tumor-associated humoral immunity

To fully harness humoral immunity in the tumor microenvironment (TME), it is critical to delineate the intracellular signal transduction cascades that govern B-cell activation and antibody-mediated effector functions. These pathways offer highly druggable nodes for targeted cancer therapy.^17,58,129^

### B-cell receptor (BCR) signaling and modulation

Within tertiary lymphoid structures (TLS), BCR signaling supports B-cell survival, proliferation, and differentiation, including germinal-center-like reactions that underpin somatic hypermutation and class-switch recombination (CSR).^17,21,130^

Upon encountering tumor-associated antigens, BCR cross-linking induces phosphorylation of immunoreceptor tyrosine-based activation motifs (ITAMs) within the Igα/Igβ signaling subunits by Src-family kinases such as Lyn, recruiting and activating spleen tyrosine kinase (Syk) as a proximal signal amplifier.^129,130^

Syk activates Bruton’s tyrosine kinase (BTK) and PI3Kδ, leading to phospholipase C gamma 2 (PLCγ2) activation, intracellular calcium release, and diacylglycerol (DAG) production; downstream MAPK, PI3K/AKT, and NF-κB signaling then drives proliferation and differentiation programs. In hematologic malignancies, BCR pathway activity is frequently sustained and provides an established therapeutic dependency.^129,130^

Accordingly, BTK inhibitors (ibrutinib,^131^ acalabrutinib^132^) and PI3Kδ inhibitors (idelalisib^133^) can silence key BCR survival signals in B-cell malignancies. In solid tumors, however, where TLS-associated BCR activation can accompany productive local immunity, pathway inhibition should be approached cautiously and ideally guided by spatial and phenotypic biomarkers that distinguish effector versus regulatory B-cell programs.^17,21^

### Fc receptor signaling dynamics: activating vs. inhibitory cascades

Antibody effector functions, including antibody-dependent cellular cytotoxicity (ADCC) and antibody-dependent cellular phagocytosis (ADCP), are dictated by engagement of Fc receptors (FcRs) on myeloid cells and natural killer (NK) cells. The balance of activating versus inhibitory FcR signaling therefore acts as a primary contextual switch in the TME.^57,58,64^

#### Activating FcRs (e.g., FcγRIII/CD16, FcγRI/CD64)

Cross-linking by clustered IgG immune complexes (often IgG1 or IgG3) phosphorylates ITAMs within FcR cytoplasmic domains or associated γ-chains, recruiting Syk to initiate signaling through PI3K and Vav. This program drives cytoskeletal remodeling required for phagocytosis and supports MAPK/ERK-dependent effector outputs including ADCP and cytotoxic granule release (ADCC).^57–59,72^

#### Inhibitory and paradoxical signaling

Tumor milieus can skew Fc signaling toward immunosuppression. The inhibitory receptor FcγRIIB signals through an immunoreceptor tyrosine-based inhibitory motif (ITIM) and, upon co-ligation, recruits phosphatases such as SHIP-1 and SHP-1 to counteract PIP3 accumulation and downstream kinase activation, thereby blunting activating Syk/PI3K cascades. In addition, in IL-10/TGF-β-rich TMEs, monomeric IgA can engage FcαRI/CD89 to deliver inhibitory ITAM (ITAMi) signaling with SHP-1 recruitment, suppressing cytotoxic myeloid effector functions.^58,64,77^

### Immunometabolic constraints on B-cell fate in the TME

Immunometabolism provides an additional layer of regulation for tumor-associated B-cell states. Antigen-activated and germinal-center-like B cells require rapid biosynthesis, redox control, and mitochondrial adaptation to support proliferation, AID activity, somatic hypermutation, and class switching. Plasma cells require sustained nutrient uptake and endoplasmic-reticulum stress adaptation for continuous antibody secretion, whereas memory B cells must preserve metabolic flexibility for rapid recall. In the hypoxic and nutrient-depleted TME, these requirements can determine whether TLS-resident B cells persist as effector/memory populations or collapse into dysfunctional states.^134,135^

Regulatory B cells and suppressive plasma-cell states may exploit distinct metabolic programs. IL-10-producing B cells have been linked to oxidative phosphorylation, cholesterol metabolism, and lipid-derived intermediates that support regulatory cytokine production, while tumor-derived lactate, hypoxia, and nutrient competition can reinforce suppressive myeloid and lymphoid states. Therapeutic manipulation of B-cell metabolism should therefore be approached cautiously: blocking metabolic pathways may impair regulatory B cells in one context but also damage germinal-center-like TLS responses or plasma-cell antibody production in another. Metabolic biomarkers should be integrated with TLS maturity, isotype, Fc-receptor, and spatial readouts before metabolic targeting is incorporated into humoral-immunotherapy trials.^134–136^

### Direct tumor cell signaling

Emerging evidence indicates that antibodies can directly engage Fcγ receptors expressed by tumor cells and activate tumor-intrinsic growth programs. In glioblastoma, plasma cell-derived IgG has been shown to bind FcγRIIA on glioma stem cells, triggering an FcγRIIA-AKT-mTOR signaling axis that sustains tumor stemness and progression.^31^

**Fig. 4:**
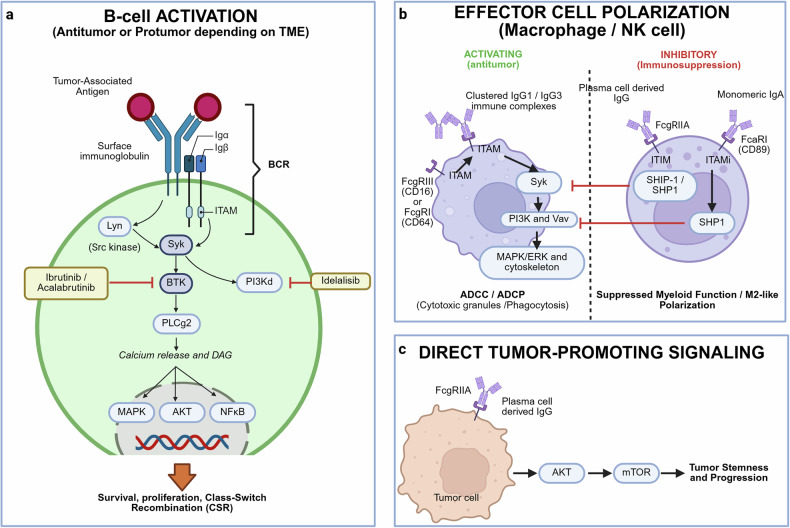
Molecular signal transduction networks governing humoral immunity in the tumor microenvironment. **a** Tumor-associated antigen-driven BCR cross-linking activates Lyn/Syk and downstream BTK, PI3K-delta, and PLC-gamma 2, triggering calcium/DAG signaling and MAPK, AKT, and NF-kappa B pathways that support B-cell survival, proliferation, and class-switch recombination; druggable nodes include BTK and PI3K-delta. **b** Activating Fc-gamma receptors signal through ITAM/Syk/PI3K/Vav to promote ADCC and ADCP, whereas inhibitory Fc-gamma RIIB and Fc-alpha RI ITAMi signaling recruit SHIP/SHP pathways that suppress myeloid effector function. **c** In glioblastoma models, plasma-cell-derived IgG can engage tumor-cell Fc-gamma RIIA and activate AKT-mTOR signaling linked to stemness and progression. The schematic was redrawn manually using non-generative vector graphics and exported as a 300 dpi JPG; no generative-AI image tool was used. Created with BioRender.com

These molecular modules are summarized in Fig. 4.

## Therapeutic targets and clinical advances in targeting humoral immunity

The interplay between humoral immunity and cancer progression suggests numerous points of therapeutic intervention. Broadly, strategies fall into two categories: (1) enhancing beneficial humoral immune responses (e.g., augmenting antitumor antibodies or supporting TLS-linked B-cell activity when it is protective), and (2) inhibiting or reprogramming harmful humoral elements (e.g., depleting immunoregulatory B-cell programs or mitigating suppressive isotype skews such as IgA/IgG4 in biomarker-defined settings). In recent years, several therapies have emerged or are in development that target B cells, antibodies, or their downstream effector pathways. Table 2 at the end of this section summarizes key FDA-approved drugs and clinical trial agents in this space across cancer types.

**Table 2:** Selected therapies targeting humoral immunity pathways in cancer

Strategy/Target	Agent (Type)	Indication (Cancer Type)	Status/Outcome
**B cell depletion**	Rituximab, anti-CD20 mAb (IgG1)	NHL, CLL (B-cell lymphomas/leukemia)	FDA-approved (1997); improved outcomes in follicular lymphoma^225^
	Obinutuzumab, glycoengineered anti-CD20	CLL, follicular lymphoma	FDA-approved (2013-2014); improved outcomes in follicular lymphoma^141^
	Ofatumumab, fully human anti-CD20	CLL	FDA-approved (2009); for refractory CLL^142^
**Plasma cell targeting**	Daratumumab, anti-CD38 mAb	Multiple myeloma	FDA-approved (2015); high response rates in multiple myeloma and related plasma-cell disorders^152,153,155^
	Elotuzumab, anti-SLAMF7 mAb	Multiple myeloma	FDA-approved (2015, combination therapy); improves outcomes in myeloma^154^
**CAR T-cell therapy**	Tisagenlecleucel, CD19 CAR-T	ALL, DLBCL (pediatric ALL, adult lymphoma)	FDA-approved (2017); first approved CAR-T therapy; high remission rates in B-ALL^147,226^
	Axicabtagene ciloleucel, CD19 CAR-T	DLBCL, high-grade B-cell lymphoma	FDA-approved (2017); high CR rates in refractory large B-cell lymphoma^149^
	Lisocabtagene maraleucel, CD19 CAR-T	DLBCL	FDA-approved (2021); active in relapsed/refractory large B-cell lymphoma^150^
	Idecabtagene vicleucel, BCMA CAR-T	Multiple myeloma	FDA-approved (2021); high response rates in refractory multiple myeloma^151^
**CAR Macrophages**	CT-0508, HER2-targeted CAR macrophage	HER2-overexpressing cancers	Phase 1; first-in-human CAR-macrophage trial in advanced solid tumors^227^
**Bispecific T-cell engagers**	Blinatumomab, CD3×CD19 BiTE	ALL (B-ALL)	FDA-approved (2014); effective in relapsed/refractory B-ALL^209,210^
	Teclistamab, CD3×BCMA bispecific Ab	Multiple myeloma	FDA-approved (2022; accelerated approval); ~65% response in heavily pretreated myeloma^211,228^
	Various (CD3×EGFR, CD3×PSMA, etc.)	Solid tumors (e.g., EGFR+ and prostate cancers)	Preclinical exploration; activity shown in EGFR- and PSMA-targeted formats^212,229^
**BCR signaling inhibitors**	Ibrutinib, BTK inhibitor (small molecule)	CLL, mantle cell lymphoma, Waldenström’s	FDA-approved (2013); high efficacy with durable disease control in B-cell malignancies^129,131^
	Acalabrutinib, zanubrutinib, BTK inhibitors	CLL, mantle cell, etc.	FDA-approved; more selective BTK inhibitors with established CLL activity^132,156^
	Idelalisib, PI3Kδ inhibitor	CLL, follicular lymphoma	FDA-approved (2014); active in relapsed CLL and follicular lymphoma^133^
	Umbralisib (PI3Kδ/CK1ε inhibitor)	Marginal zone & follicular lymphoma	Previously reported phase 2 activity; FDA approval withdrawn in 2022 because of safety concerns^230,231^
**Checkpoint inhibitors**	Ipilimumab, anti-CTLA-4 mAb	Melanoma (also some others in combination)	FDA-approved (2011); first CTLA-4 checkpoint inhibitor in oncology^177–179^
	Nivolumab, pembrolizumab, anti-PD-1	Melanoma, NSCLC, RCC, etc.	FDA-approved (2014 onward); broad activity across multiple solid tumors^179,232–234^
	Atezolizumab, durvalumab, anti-PD-L1	NSCLC, bladder, etc.	FDA-approved (2016 onward); established combination use in solid tumors^235^
**Cancer vaccines (humoral focus)**	Sipuleucel-T (Provenge), PAP GM-CSF cell vaccine	Prostate cancer (metastatic)	FDA-approved (2010); modest survival benefit in metastatic CRPC^236^
	NeuVax (nelipepimut-S), HER2 peptide vaccine	Breast cancer (HER2 low)	Phase 3 did not meet its primary endpoint in breast cancer^237^
	mRNA neoantigen vaccines	Melanoma, etc. (experimental)	Early studies show immunogenicity; phase 2b benefit reported in melanoma^183,184^

This table is not exhaustive, but it highlights the breadth of approaches targeting humoral components in cancer therapy

### Targeting B cells and plasma cells

One direct way to modulate humoral immunity in cancer is to eliminate or alter the B cells themselves:

#### Anti-CD20 monoclonal antibodies

CD20 is expressed on most B cells (except pro-B and plasma cells). Rituximab, a chimeric IgG1 anti-CD20, was a game-changer in B-cell non-Hodgkin lymphomas (approved 1997).^137–139^ It mediates B cell depletion via ADCC, complement, and apoptosis induction, leading to high response rates in lymphomas when combined with chemotherapy.^137–139^ Following rituximab,^140^ humanized and fully human anti-CD20s (obinutuzumab,^141^ ofatumumab^142^) were developed and are FDA-approved for CLL and follicular lymphoma. These drugs unequivocally demonstrate the benefit of removing B cells in B-cell malignancies. Interestingly, in solid tumors, anti-CD20 has also been tested to remove B cells in the tumor microenvironment if they are thought to be pro-tumor.^143,144^ A small trial of rituximab in metastatic melanoma (intending to deplete TLS B cells) showed some reduction in immunosuppressive cytokines but didn’t significantly improve outcomes; more research is needed to identify contexts where depleting B cells in solid tumors is beneficial versus harmful (since B cells can also be tumor-fighting).^145^

#### Anti-CD19 and anti-BCMA therapies

CD19 is a pan-B marker (expressed from early development through mature B cells and lost upon plasma cell differentiation) and is also present on some plasmablasts. The rise of chimeric antigen receptor (CAR) T-cell therapy targeting CD19 has revolutionized relapsed B-cell acute lymphoblastic leukemia (ALL) and aggressive lymphomas.^146,147^ CAR T cells (tisagenlecleucel,^148^ axicabtagene ciloleucel,^149^ lisocabtagene maraleucel^150^) eliminate CD19⁺ cells, inducing high remission rates in refractory cases. By wiping out malignant (and normal) B cells, they remove the source of pathogenic Ig in these cancers (in ALL, the Ig itself is not the issue, but the malignant B cell is). In multiple myeloma (a plasma cell cancer), CAR T cells against BCMA (B-cell maturation antigen), a plasma cell marker, have shown high efficacy (idecabtagene vicleucel approved in 2021).^151^ These represent a strategy of targeting the humoral immune cell origin to treat the cancer.

#### Anti-CD38 and anti-SLAMF7

Plasma cell-directed mAbs such as daratumumab^152,153^ (anti-CD38) and elotuzumab^154^ (anti-SLAMF7) are approved in multiple myeloma. Daratumumab depletes plasma cells by ADCC and complement and has become a backbone of myeloma therapy, significantly extending patient survival.^152,153,155^ While these therapies are aimed at malignant plasma cells, an interesting thought is whether similar approaches can target long-lived plasma cells producing pro-tumor antibodies in the microenvironment of solid tumors (though identifying such cells specifically is challenging).

#### Bruton Tyrosine Kinase (BTK) Inhibitors

BTK is crucial in B-cell receptor signaling. In CLL and mantle cell lymphoma, BTK inhibitors like ibrutinib,^131^ acalabrutinib,^132^ and zanubrutinib^156^ have transformed treatment by effectively silencing BCR-driven survival signals, leading to apoptosis of malignant B cells. These are small molecules (oral drugs) and represent targeted therapy that disrupts humoral signaling. There is interest in exploring BTK inhibitors in solid tumors where B cells or even tumor cells’ BCR-like signaling can play a role. As of now, BTK inhibitors have not shown clear utility in solid tumors, but trials are ongoing in cancers like pancreatic and colon cancer where B cell-rich stroma is present. Additionally, BTK is expressed in myeloid cells,^157^ so BTK inhibitors can also dampen certain myeloid-driven inflammation (which can be beneficial in cancers with an inflammatory microenvironment).

#### PI3K inhibitors (Idelalisib, etc.)

PI3Kδ is primarily expressed in B cells. Idelalisib^133^ (a PI3Kδ inhibitor) was approved for CLL and follicular lymphoma as it induces B-cell death by blocking downstream BCR and cytokine signaling. Its use has been hampered by immune-related toxicities (colitis, etc.), showing the delicate balance when targeting B-cell pathways that also exist in normal immune cells.

#### Targeting regulatory B cells

There is no specific approved therapy yet for Bregs, but preclinical studies show that IL-10 or PD-L1 blockade can disrupt Breg function.^158–160^ Since Bregs often express PD-L1, checkpoint inhibitors like anti-PD-L1 can also act on those Bregs, not just on tumor or myeloid cells. Some explorations involve using anti-CD25 (usually aimed at Tregs) to see if Bregs (which can express CD25 in mice) are affected.^161,162^

#### BAFF/APRIL pathway inhibition

BAFF and APRIL are cytokines that promote B cell survival and plasma cell generation.^163,164^ Belimumab (anti-BAFF) is used in lupus to tone down B cells.^165^ BAFF/APRIL biology links B-cell survival to cancer-associated inflammatory programs.^166^ A trial of BAFF blockade in follicular lymphoma did not yield much benefit, because malignant B cells are autonomous. In solid tumors, BAFF levels can correlate with TLS formation (BAFF is often produced in TLS), but blocking it can hamper anti-tumor B cells more than help.^167^

In essence, depleting or inhibiting B cells is very effective for B-cell cancers and is being carefully considered for scenarios in solid tumors where B cells are pathological (e.g. in virally driven tumors where B cells can promote immune tolerance).^129,168–170^ More frequently, though, for solid tumors, the trend is to enhance B cells, especially those forming TLS, because they correlate with good outcomes.^171,172^ This divergence makes the therapy approach context-dependent. For example, intratumoral administration of a Toll-like receptor 9 (TLR9) agonist (CpG) has been used to spark TLS formation and enhance local B cell-driven immune activity in melanoma, with encouraging immune activation in early trials.

### Modulating antibody effector biology (Fc receptors, complement, IgG4/IgA)

Antibody effector pathways can be therapeutically tuned in solid tumors, either to enhance productive Fc-mediated tumor clearance or to mitigate immunosuppressive isotypes and chronic immune-complex effects. Because these pathways are shared with host protective immunity, clinical translation requires context-specific guardrails and biomarker-guided patient selection.

#### Fc receptor blockade/engineering

Many approved therapeutic antibodies already exploit Fcγ receptor engagement, and next-generation approaches further tune Fc activity (e.g., Fc engineering to enhance activating FcγR binding or reduce inhibitory FcγRIIb engagement) in a context-dependent manner.^58,137–139,173^

#### Complement modulation

Complement can contribute to antibody efficacy but can also promote chronic inflammation and immune suppression depending on context. Clinically available complement inhibitors (e.g., anti-C5 eculizumab^174^) highlight feasibility, whereas tumor-specific strategies aim to overcome complement regulators (CD46, CD55, CD59) to enable complement-dependent cytotoxicity.^175,176^ Given potential systemic toxicity, such strategies require careful patient selection and monitoring.

#### IgG4 and IgA axis targeting

To counter IgG4-associated immune dampening, strategies under exploration include Fc–Fc blocking peptides and Fc engineering to prevent inhibitory Fc engagement or Fab-arm exchange.^173^ For IgA, the goal is to distinguish suppressive IgA⁺ regulatory plasma-cell programs from cytotoxic antigen-specific IgA immune complexes; this can involve targeting upstream regulatory cytokines (e.g., TGF-β) or leveraging engineered IgA therapeutics in settings where neutrophil effector activity can be productively engaged.

### Immune checkpoint inhibitors and vaccines

While immune checkpoint inhibitors (ICIs), such as anti-PD-1/PD-L1 and anti-CTLA-4 agents, primarily reinvigorate T cells, they also affect B cells and humoral responses.^177–179^ For instance, anti-CTLA-4 can promote formation of TLS and B-cell responses in melanoma (since CTLA-4 is expressed on T follicular helper cells that regulate B cells). Additionally, PD-1 is expressed on B cells, especially in germinal centers; PD-1 blockade can enhance antibody production or class switching in those TLS.^16,27^ Indeed, patients responding to ICIs often show increased antibody titers to tumor antigens. ICIs are now standard of care in many cancers and can synergize with strategies that target humoral immunity by freeing B cells from PD-1/PD-L1 inhibition.^180,181^

Checkpoint blockade also has a humoral double-edged effect. By releasing PD-1/PD-L1 or CTLA-4 constraints, ICIs can strengthen Tfh-B-cell crosstalk, TLS maturation, and tumor-specific antibody responses; however, the same process can expand autoreactive B-cell clones, increase plasmablast activity, and contribute to immune-related adverse events. Trial designs that combine ICIs with B-cell-modulating agents should therefore include baseline autoantibody assessment, longitudinal B-cell subset profiling, and predefined toxicity algorithms, particularly when CTLA-4 and PD-1 blockade are combined.^178,179,182^

Cancer vaccines aimed at inducing antibody responses have historically had limited success, but new vaccine platforms (mRNA vaccines, vector-based vaccines) are being tested to generate both T cell and B cell immunity against tumor antigens. For example, an mRNA vaccine encoding neoantigens in melanoma induced not just T cell responses but also antigen-specific antibody responses.^183,184^ While the T cell effect can dominate tumor killing, antibodies can contribute by opsonizing tumor cells or neutralizing growth factors. An interesting vaccine approach is idiotype vaccination in B-cell lymphomas, where patients were immunized with a protein mimicking the unique Ig of their tumor (to raise anti-idiotype antibodies).^185–187^ Despite immunogenicity, phase III trials did not significantly improve outcomes (due to tumor immune evasion), and this approach was largely abandoned for lymphomas in favor of passive immunotherapy like rituximab. In solid tumors, efforts to develop vaccines that generate high-affinity IgG (for example, anti-HER2 vaccines to induce something akin to trastuzumab) are ongoing.^188^ Some patients do develop such antibodies spontaneously or via vaccination, but achieving therapeutic titers has been challenging. Nonetheless, as checkpoint blockade provides a more permissive immune environment, combining vaccines (to induce humoral and cellular responses) with ICIs is an active area of trials (e.g., vaccines in triple-negative breast cancer to induce anti-MUC1 antibodies alongside pembrolizumab).^189,190^

### CAR-T and innate-cell approaches leveraging humoral specificity

Beyond CAR-T cells against B cell markers,^191,192^ a new wave involves CAR-T cells using B cell–derived antigen receptors as targeting moieties for solid tumors.^193,194^ For example, researchers have isolated a high-affinity antibody (from a B cell) that binds a tumor antigen and then used its scFv in a CAR construct.^195^ Many CAR-T therapies for solid tumors (HER2 CARs, EGFRvIII CARs, etc.) essentially transfer humoral specificity into T cells.^196^ This is indirectly leveraging humoral immunity by borrowing antibody targeting. The success of CAR-T in solid tumors has been limited by the tumor microenvironment, but the concept remains promising. CAR-Macrophages and other innate immune cell-based CAR strategies have also emerged for treating solid tumors.^197–206^ These approaches use engineered innate immune cells to attack tumors. In one approach, CAR-Mφ were designed to phagocytose tumor cells opsonized by antibodies. They showed high potential in preclinical models by resisting the tumor’s immunosuppressive signals and actively eating tumor cells. Another innovative concept is “NKT-CAR” cells, Natural Killer T cells engineered with CARs, being tested for solid tumors.^207,208^

### Bispecific T-cell engagers (BiTEs)

Like blinatumomab (anti-CD3 × anti-CD19 for ALL), they are another example where an antibody-based construct brings T cells to tumor cells.^209,210^ A BiTE essentially mimics the synapse that a B cell–derived antibody can create, directing T cells to kill target cells. Now, bispecifics are being developed for various targets: e.g., CD3 × BCMA (teclistamab, for myeloma^211^), or CD3 × EGFR (in trials for solid tumors).^212^ These belong to the antibody therapy arsenal, extending humoral targeting to engage cellular immunity.

### Enhancing beneficial humoral responses and inducing TLS

Beyond depleting deleterious humoral elements, several strategies boost antitumor B-cell immunity or promote TLS formation: TLR9 agonists (CpG ODNs) activate B cells and plasmacytoid dendritic cells, enhance Th1-skewing, and function as vaccine adjuvants; early trials and combinations support feasibility and immunologic activity.^171,172^ TLS induction approaches leverage CXCL13/CCL19/CCL21 gradients and LTβR/LIGHT signaling; STING agonism and biomaterial-based delivery can drive intratumoral CXCL13/CCL19/CCL21 and HEV/TLS formation in vivo, improving antitumor immunity in models.^86,87^ These modalities complement T-cell–directed therapies and aim to enhance B cell–derived antibody quality (mature TLS) rather than indiscriminate B-cell depletion.

### Therapeutic timing: neoadjuvant versus advanced-disease humoral modulation

B-cell expansion and TLS formation are often more interpretable, and potentially more robust, in the neoadjuvant setting because the intact tumor supplies abundant antigen, preserved stromal scaffolds, draining-lymphatic connections, and spatially measurable immune niches. Short-course neoadjuvant therapy also enables paired pretreatment and on-treatment tissue sampling, allowing investigators to detect early CXCL13 induction, HEV/FDC maturation, Tfh-B-cell proximity, BCR clonal expansion, class switching, and plasma-cell differentiation before surgical removal. In advanced or heavily pretreated disease, tumor immune editing, necrosis, fibrosis, prior chemotherapy, antibiotics, steroids, and loss of stromal organization may blunt TLS formation or bias B cells toward regulatory programs.^27,47,85,89^

This temporal asymmetry should shape clinical trials of B-cell-modulating agents. TLS-inducing or B-cell-enhancing strategies are most rational as window-of-opportunity neoadjuvant studies with mandatory spatial and repertoire endpoints. Conversely, B-cell depletion, BTK/PI3K inhibition, or IgA/IgG4-axis modulation should not be tested indiscriminately; pretreatment diagnostic panels should first distinguish TLS-mature tumors, where B-cell depletion may be harmful, from tumors dominated by diffuse IL-10+ Bregs, suppressive IgA+ plasma cells, IgG4 skewing, inhibitory Fc-receptor signaling, or absent TLS, where targeted modulation may be beneficial. These guardrails should be built into eligibility criteria, stratification, and stopping rules.

### Clinical progress and ongoing trials

Many of the concepts above are in clinical evaluation. Table 2 summarizes key agents and their status:

## A note on tumor-derived immunoglobulins (TD-Igs): a controversial axis

Beyond the well-established contributions of B-lineage cells, a provocative and still debated literature proposes that some non-hematopoietic tumor cells can themselves express immunoglobulin transcripts and/or proteins. Reports span breast, colon, lung, liver, and oral epithelial tumors, with additional claims in other malignancies, but the field remains conceptually and technically unsettled.^213–218^ It is therefore essential to distinguish putative tumor-derived immunoglobulins (TD-Igs) from the much broader immunoglobulin superfamily (IgSF), which comprises many proteins with Ig-like domains but does not imply rearranged immunoglobulin loci.^219^ If authentic, TD-Igs would represent ectopic V(D)J recombination and, in at least some reports, class-switch recombination outside the B-cell lineage.^215–217^

### Comparison with canonical immunoglobulins

Canonical B-cell-derived immunoglobulins are full-length H2L2 glycoproteins whose effector functions are tuned by subclass, Fc receptor engagement, and the conserved IgG Fc N-glycan at Asn297.^52–54,82^ In contrast, the TD-Ig literature describes molecules with several non-canonical features, including heavy-chain-dominant or otherwise structurally atypical species, variable evidence for complete heavy-light pairing and secretion, and glycoforms proposed to differ from conventional B-cell IgG.^217–219^ In RP215-based studies, a sialylated N-glycan centered on Asn162 in the CH1 region has been proposed as a cancer-associated feature, and additional O-glycans have also been reported.^61,217–219^ Importantly, peptide mapping of CA215 preparations showed that these preparations contain multiple IgSF proteins, underscoring the need for caution in equating RP215 reactivity with a fully assembled canonical immunoglobulin.^219^

### Exemplar models: integrin/FAK and c-Met-SOX2-IgG loops

Two widely cited examples illustrate both the interest of the field and the narrowness of the evidence base. In lung squamous cell carcinoma cell lines, non-canonically glycosylated IgG was reported to engage integrin alpha6beta4 and activate FAK-dependent survival and migration programs.^220^ In lung adenocarcinoma models, a self-propagating c-Met-SOX2-IgG loop was reported to sustain stem-like properties and tumor growth.^221^ Both mechanisms have so far been supported mainly in selected cell lines and xenograft systems rather than by broad, independently replicated demonstrations in primary human tumors.^217,218^

### State of the evidence

A critical appraisal suggests that most mechanistic TD-Ig literature remains Evidence Tier C, dominated by cell lines, xenografts, and bulk-tissue assays that are vulnerable to confounding.^213–218^ Even when IG transcripts or protein are detected in tumor specimens, stringent exclusion of infiltrating B cells or plasma cells, serum entrapment, extracellular uptake, and Fc-binding artefacts is often incomplete.^216–219^ Claims from single-cell RNA-seq additionally require protection against ambient RNA and doublets, and long-term cultured cell lines require special caution because ongoing genetic and transcriptional drift can materially alter phenotypes.^222,223^ (Box 1).

Box 1 Caveats and controversies in tumor-derived immunoglobulin research

- **Experimental pitfalls - B-cell/plasma-cell contamination**: bulk-tissue detection of IGH/IGK/IGL transcripts or immunoglobulin protein does not establish synthesis by malignant cells; claims require cell-type-resolved localization and, ideally, paired DNA/RNA/protein evidence in the same population.^214–217^
- **Experimental pitfalls - Uptake versus synthesis**: cancer cells can accumulate extracellular proteins and can also be associated with exosomal cargo; endogenous synthesis should be supported by evidence of nascent biosynthesis and intracellular processing rather than surface staining alone.^219,238^
- **Experimental pitfalls - Cell line drift:** long-term cultured lines can evolve genetically and transcriptionally, so replication across independent models and validation in primary tissue are essential.^223^
- **Experimental pitfalls - Fc-binding artefacts and reagent specificity:** Fc-binding proteins and incompletely validated antibodies can generate false positives, and RP215-reactive CA215 preparations contain multiple IgSF proteins rather than a single canonical immunoglobulin species.^219^
- **Alternative explanations - Ambient RNA in single-cell sequencing:** low-level IG reads in malignant cells can reflect ambient RNA or doublets unless dedicated decontamination and stringent gating are applied.^222^
- **What is needed - Multimodal validation**: co-detection of rearranged IG loci in purified malignant cells, transcripts by spatially resolved methods, and protein with well-validated reagents, coupled to in vivo genetic perturbation and independent multi-laboratory replication.^215–218^

### Clinical implications

At present, TD-Igs are not established clinical biomarkers or therapeutic targets. RP215-based strategies and related approaches remain preclinical concepts, and no validated diagnostic assay or approved drug currently depends on TD-Ig targeting.^217,218,224^ Any future clinical translation will require robust evidence of prevalence, tumor-cell specificity, functional necessity, and safety, particularly because interventions touching immunoglobulin biology risk collateral effects on normal humoral immunity.^217,218^

In summary, TD-Igs remain a fascinating but unproven axis of cancer biology. At present, they should be interpreted separately from the established roles of B-cell-derived antibodies in antitumor and protumor immunity, and any generalization beyond well-controlled exemplar studies would be premature.^213–219^

## Conclusions and Perspectives

Humoral immunity in cancer should be viewed as a spatially organized, state-dependent immune program rather than as a uniform B-cell signature. The strongest antitumor configuration is emerging as a mature TLS-centered ecosystem: high endothelial venules recruit lymphocytes; follicular dendritic-cell networks and CXCL13 gradients retain CXCR5+ B cells and Tfh cells; IL-21 and CD40L sustain germinal-center-like selection; and clonally expanded, somatically mutated, class-switched B cells generate plasma cells and memory B cells. In this setting, B cells can present antigen, reinforce CD4+ and CD8+ T-cell activity, and produce IgG1/IgG3-dominant or otherwise productive antibodies that recruit ADCC, ADCP, and complement. BCR clonality is therefore most informative when interpreted as evidence of antigen-driven selection, particularly when coupled to somatic hypermutation, class-switching, phenotype, and spatial localization inside TLS. Conversely, humoral infiltration outside mature TLS may reflect diffuse regulatory programs, bystander activation, serum immunoglobulin extravasation, or chronic immune-complex biology rather than effective tumor recognition. This distinction is central for clinical interpretation because a high B-cell score can either mark a coordinated immune attack or a suppressive niche, depending on the cellular state and tissue architecture.

The major limitation for the field is that the same molecules can have opposite consequences depending on anatomical and biochemical context. IgA-rich mucosal tumors may contain cytotoxic antigen-specific IgA immune complexes, but IgA+ regulatory plasma cells in IL-10/TGF-beta-rich niches can suppress CD8+ T cells and bias myeloid FcαRI signaling toward ITAMi pathways. IgG4 can mark chronic antigen exposure and may competitively blunt IgG1-mediated Fc effector activity, yet subclass abundance alone does not prove function without antigen specificity, immune-complex geometry, Fc-receptor mapping, and functional readouts. Complement can assist antibody-mediated tumor clearance but can also sustain inflammation when clearance is incomplete. Immunometabolism adds another constraint: germinal-center-like B cells, memory B cells, plasma cells, and IL-10-producing regulatory B cells differ in their reliance on glycolysis, oxidative phosphorylation, lipid and cholesterol metabolism, and their ability to survive in hypoxic, nutrient-depleted tumor regions. These variables explain why pan-cancer associations are useful for hypothesis generation but insufficient for prescribing B-cell depletion or augmentation without tumor-specific biomarker validation. A key challenge is to convert descriptive atlases into causal models that predict which humoral states can be safely amplified and which should be selectively restrained.

Clinical translation should therefore proceed through prescriptive guardrails. Trials that enhance humoral immunity should preferentially enroll or stratify TLS-mature, immune-inflamed tumors and should measure TLS density, HEV and FDC networks, GC-like zones, Tfh-B-cell proximity, BCR clonality, SHM burden, class-switching status, isotype/subclass distribution, Fc-receptor balance, and Fc glycofeatures before and during therapy. In contrast, trials testing B-cell depletion, BCR-pathway inhibition, or IgA/IgG4-axis modulation should be restricted to tumors in which pretreatment diagnostics show diffuse regulatory B-cell infiltrates, IgA+ or IgG4-skewed suppressive plasma-cell states, inhibitory FcγRIIB/FcαRI signaling, or absence of mature TLS. Neoadjuvant studies are especially attractive because the intact tumor supplies antigen, stromal scaffolds, and spatially measurable immune niches; paired pretreatment and on-treatment specimens can identify early B-cell expansion, TLS maturation, and humoral memory formation before systemic therapy, surgery, or advanced immune editing erodes these structures. This temporal asymmetry should guide window-of-opportunity designs and prevent indiscriminate B-cell modulation in settings where TLS are already clinically beneficial.

The next methodological priority is integration. TLS maturity metrics should be standardized across tumor types, but a histological TLS score alone is not enough. Spatial transcriptomics can define chemokine and lymphocyte neighborhoods, spatial proteomics can quantify receptor-ligand and Fc-receptor states at single-cell resolution, and paired scRNA-seq/scBCR-seq can link each B-cell clone to phenotype, isotype, SHM, and class-switching. Integration of spatial proteomics with scBCR-seq is particularly valuable for refining the TLS maturity metrics mentioned in this review because it can determine whether expanded clones occupy GC-like TLS, whether plasma cells emerge along TLS-to-margin trajectories, and whether regulatory B cells are dispersed in hypoxic or myeloid-rich stroma. Standardized pipelines should also distinguish true tumor-cell-associated immunoglobulin signals from B-cell contamination, ambient RNA, serum leakage, or Fc-binding artifacts, particularly in CNS tumors with blood-brain barrier disruption and in studies of proposed tumor-derived immunoglobulins.

Therapeutically, the most rational future is not simply to add or remove B cells, but to tune humoral immunity according to the pretreatment ecosystem. TLS-inducing strategies, in situ vaccination, STING or lymphotoxin-axis activation, CpG/TLR agonists, vaccines, and Fc-engineered antibodies may be most useful when they mature existing immune scaffolds and favor productive class-switched effector states. Conversely, selective disruption of IL-10/TGF-beta-driven Bregs, suppressive IgA+ plasma cells, IgG4-biased circuits, inhibitory Fc-receptor signaling, or pathogenic BCR-pathway dependencies may benefit patients whose tumors lack mature TLS and are dominated by regulatory humoral infiltrates. Immune checkpoint inhibitors require a similar balance: they can amplify Tfh-B-cell crosstalk and antibody responses, but they can also release autoreactive B-cell clones and contribute to immune-related adverse events. Future trials should therefore combine efficacy endpoints with safety monitoring of autoantibodies, B-cell subsets, infection risk, and humoral memory. The final goal is a classification in which each patient is assigned a humoral immune contexture, such as TLS-mature effector, mixed TLS-regulatory, diffuse Breg-dominant, IgA/IgG4-skewed, or humoral-cold, and treated with matching interventions. If these guardrails are implemented, humoral immunity can become a clinically actionable dimension of precision immuno-oncology, complementing T-cell biomarkers while preserving the protective functions of mature TLS. Future studies should also predefine minimum reporting sets for humoral biomarkers: how TLS maturity was scored, how BCR clonotypes were called, which isotype/subclass and Fc-glycan assays were used, where B-cell subsets were positioned relative to tumor nests and vessels, and how safety endpoints such as infection, hypogammaglobulinemia, autoantibodies and irAEs were monitored. Harmonized reporting will make trials comparable across tumor types and will help distinguish biologically meaningful negative results from failures caused by undersampling, inconsistent tissue processing or non-equivalent definitions of TLS maturity.

## Supplementary information

Table S1

## Data Availability

No new datasets were generated or analyzed for this review article.

## **Author’s Note on AI**

The authors used DeepL Pro solely to improve language clarity. No generative-AI tool was used to generate or modify figures; all figures were redrawn manually as non-generative vector schematics and reviewed by the authors for accuracy, interpretation, and consistency with the text and legends.
