# Supplementary material for: Humoral immunity in cancer
Source: Signal Transduct Target Ther. 2026 Aug 19;11:335. doi: 10.1038/s41392-026-02902-2 (PMC13486706; doi:10.1038/s41392-026-02902-2)
Supplement: Supplementary file 1 [file 41392_2026_2902_MOESM1_ESM.docx]

**Table S1:** Cross-cancer comparison of humoral features highlighted in this review (illustrative examples). Columns summarize TLS organization, dominant B/plasma states, isotype/subclass skews, and reported clinical associations. Abbreviations used in the table: TNBC, triple-negative breast cancer; NSCLC, non-small-cell lung cancer; CNS, central nervous system; GBM, glioblastoma; BBB/BTB, blood-brain barrier/blood-tumor barrier; ICI, immune checkpoint inhibitor; GC, germinal center; TLS, tertiary lymphoid structure; HEV, high endothelial venule; FDC, follicular dendritic cell; Tfh, T follicular helper cell; IL-10, interleukin-10; TGF-β, transforming growth factor beta; PD-L1, programmed death ligand 1; Ig, immunoglobulin; Bregs, regulatory B cells; TAMs, tumor-associated macrophages; ADCC, antibody-dependent cellular cytotoxicity; ADCP, antibody-dependent cellular phagocytosis.

| Cancer context | TLS prevalence / maturity | Dominant B/plasma states | Notable isotype/subclass features | Reported associations / notes |
| --- | --- | --- | --- | --- |
| Breast cancer (esp. TNBC) | TLS are common in subsets; GC-like maturity varies across cohorts | GC-like B cells and class-switched plasma cells in TLS; context-dependent Breg niches | IgG class switching; occasional IgG4 in chronic regulatory niches | TLS signatures often associate with improved prognosis and ICI benefit; regulatory programs can emerge in inflamed settings |
| Lung cancer (NSCLC) | TLS frequently observed in resected tumors; maturity correlates with immune infiltration | Memory/GC-like B cells and plasma cells; IgA⁺ regulatory plasma cells reported in subsets | IgA and IgG coexist; IgA programs can be suppressive or cytotoxic depending on immune-complex context | TLS maturity associates with better outcome/ICI response in multiple studies; IgA⁺ IL-10/PD-L1 programs may counteract immunity |
| CNS tumors (GBM/brain metastases) | TLS-like aggregates reported but variable; BBB disruption influences exposure to serum Ig | Intratumoral B cells/plasma cells with immunoregulatory signatures in some cohorts | Serum Ig extravasation plus local plasma-cell Ig likely dominate tissue Ig pools | Antibody-associated phenotypes should be interpreted considering BBB/BTB status, vascular leak, and intratumoral B/plasma-cell burden. |
